# Increased healthcare utilization associated with complete atrioventricular block in pacemaker patients

**DOI:** 10.1007/s10840-018-0336-0

**Published:** 2018-02-28

**Authors:** Suneet Mittal, Dan L. Musat, Michael H. Hoskins, Julie B. Prillinger, Gregory J. Roberts, Yelena Nabutovsky, Faisal M. Merchant

**Affiliations:** 1 The Snyder Center for Comprehensive Atrial Fibrillation, the Valley Health System, Ridgewood, NJ USA; 2 New York, USA; 3 Emory University School of Medicine, Atlanta, GA USA; 4 Abbott, Sylmar, CA USA

**Keywords:** Pacemakers, Heart failure, Atrioventricular block, Right ventricular pacing, Healthcare utilization

## Abstract

**Purpose:**

The purpose of the current study is to characterize and quantify the impact of complete atrioventricular block (cAVB) on heart failure hospitalization (HFH) and healthcare utilization in pacemaker (PM) patients.

**Methods:**

Patients ≥ 18 years implanted with a dual-chamber PM from April 2008 to March 2014 were selected from the MarketScan® Commercial and Medicare Supplemental claims databases. Patients with ≤ 1-year continuous MarketScan enrollment prior to and post-implant, and those with prior HF diagnosis were excluded. Patients were dichotomized into those with cAVB, defined as a 3rd degree AVB diagnosis or AV node ablation in the year prior to PM implant, versus those without any AVB (noAVB). Post-implant HFH and associated costs were compared based on inpatient claims.

**Results:**

The study cohort included 21,202 patients, of which 14,208 had no AVB and 6994 had cAVB, followed for 2.39 and 2.27 years, respectively. Patients with cAVB were associated with a significantly increased risk of cumulative HFH (HR 1.59 [95% CI 1.35–1.86] *p* < 0.001) and significantly higher costs ($636 [609–697] vs $369 [353–405] per pt-year, *p* < 0.001) compared to those with no AVB.

**Conclusions:**

Among dual-chamber PM patients without prior HF, cAVB is associated with a significantly increased risk of HFH and greater HF-related healthcare utilization. Identifying patients at high risk for HF in the setting of RV pacing, and potentially earlier use of biventricular or selective conduction system pacing, may reduce HF-related healthcare utilization.

**Electronic supplementary material:**

The online version of this article (10.1007/s10840-018-0336-0) contains supplementary material, which is available to authorized users.

## Introduction

Pacemaker implantation is most commonly performed in patients with symptomatic sinus node dysfunction or atrioventricular block (AVB). It is now recognized that some patients develop a pacing induced cardiomyopathy due to the dyssynchrony induced by right ventricular pacing [1, 2]. Although pacing algorithms have been developed to minimize ventricular pacing in patients with sinus node dysfunction, patients with advanced heart block require ventricular pacing. Most recent studies have focused on showing incidence of new heart failure onset associated with AV block and identifying predictors of pacing induced cardiomyopathy [1, 3–5]. However, the impact of the new heart failure onset on healthcare utilization has not been studied. Heart failure imposes an enormous burden on the healthcare system, consuming more Medicare dollars than any other diagnosis [6]. Therefore, in this large retrospective study using real-world data from a nationwide billing claims database, we sought to quantify the impact of complete AVB at the time of pacemaker implant on heart failure-related healthcare utilization. Specifically, we sought to compare incidence of heart failure hospitalizations and concomitant heart failure-related costs between patients with and without complete AVB at the time of dual-chamber pacemaker implantation. We have previously published on the clinical experience of these pacemaker patients and found an elevated risk of new HF development associated with complete AVB [1]. This work builds on the prior analysis by evaluating the associated impact to the US healthcare system.

## Methods

### Data source

Retrospective data for this study were derived from the Truven Health MarketScan® Commercial Claims and Medicare Supplemental databases, which capture paid and adjudicated billing claims from inpatient hospital encounters and outpatient physician office visits for privately insured and Medicare Supplemental patients throughout the USA. The nationally representative databases include records from > 170 million enrollees since 1995 [7] and have supported publications on outcomes of patients undergoing cardiac procedures and receiving implantable electronic devices [1, 8–10].

### Study population

Patients implanted with a *de novo* dual chamber pacemaker (*Current Procedural Terminology [CPT]* code 33208 and/or *Healthcare Common Procedure Coding System [HCPCS]* codes C1785, C2619) from any manufacturer between April 1, 2008, and March 31, 2014, were selected for study inclusion. *De novo* implants were identified in the MarketScan® databases as pacemaker (PM) patients without a prior device implant and without a remote or in-office PM follow-up visit in the 1 year prior to implant. Patients with a left ventricular lead placed (*CPT* codes 33224 or 33225) at the time of PM implant were excluded. All included patients had at least 1 year of continuous MarketScan® enrollment prior to and post-PM implant, as evidenced by a monthly enrollment indicator in the MarketScan® database. Finally, patients were required to be ≥ 18 and ≤ 100 years old at the time of PM implant and without a primary or secondary diagnosis of heart failure (HF) prior to PM implant.

To evaluate the impact of atrioventricular block (AVB) on hospitalizations following PM implant, the study cohort was dichotomized into patients with a diagnosis of complete AVB (cAVB) versus those without a diagnosis of AVB (noAVB). Patients with cAVB were identified by a diagnosis of third degree AVB (*International Classification of Diseases, Ninth Revision [ICD-9]* code 426.0) or an ablation of the atrioventricular junction (AVJ) (*CPT* code 93650) in the 1 year prior to PM implant. Patients with an AVJ ablation occurring > 1 year prior to PM implant or at any time after PM implant were excluded from the study. The noAVB cohort included patients who were never diagnosed with any degree of AVB (*ICD-9* codes 426.0–426.1) throughout the study period. Patients with cAVB were presumed to have a high burden of right ventricular (RV) pacing relative to noAVB patients, although the actual percent of RV pacing is not available in the MarketScan® databases.

Patient demographics were characterized using age, sex, remote monitoring status, US region, year of PM implant, and 20 baseline (≤ 1 year prior to implant) comorbidities based on the Charlson comorbidity index. Patients were defined as active on remote monitoring if they transmitted ≥ 1 remote follow-up within 1 year following PM implant. US regions included Northeast, North Central, South, and West. Claims codes used for diagnoses and procedures were collected across all available fields (up to 15) in the MarketScan® inpatient and outpatient encounters, as shown in the Supplement (Table S1) and validated previously [11, 12]. Propensity scores for the diagnosis of cAVB were calculated for every patient in the study cohort based on a multivariable logistic regression model including all covariates used in the patient characterization.

### Outcomes

The primary outcomes included HF hospitalizations (HFHs) and associated payments following dual-chamber PM implant. A HFH was identified in the MarketScan® databases as any inpatient encounter for which the primary diagnosis was HF-related, as outlined in the Supplement (Table S1). The unadjusted rate of HFH (events per 100 patient-years [pt-years]) was computed as the cumulative number of HFH divided by the total duration of patient follow-up for each group.

Both unadjusted (actual) and adjusted (predicted) payments associated with HFH were computed for patients with noAVB and cAVB. Due to differences in reimbursement rates and patient demographics, unadjusted payments are reported separately for patients covered by commercial insurance and those with Medicare Supplemental plans. A two-part model was utilized to predict the annual adjusted HFH payments in noAVB and cAVB patients following PM implant. The two-part model is a well-established econometric modeling technique that accounts for samples with a large proportion of zero measurements, common to healthcare data in which healthy participants incur no medical costs. Further, the model enables adjustment for patient characteristics. In part 1, a logistic regression was used to model the likelihood of incurring nonzero payments following PM implant, adjusting for AVB group, follow-up duration, and the computed propensity score. Using this model, the numeric probability of incurring nonzero payments at 1 year post-implant was then estimated for each patient. In part 2, using only those patients who had nonzero hospitalization payments following PM implant, a linear regression with gamma distribution and log link was used to model the total hospitalization costs, adjusting again for AVB group, follow-up duration, and the computed propensity score. The total payments at 1 year post-implant were then predicted for all patients using results from the linear regression model. The final adjusted HFH payment for each patient was computed as the product of the probability from part one and the predicted payments from part 2.

Secondary outcomes included the length of stay (LOS) for each hospitalization and rates of 30-day HF readmissions as defined by the Centers for Medicare and Medicaid Services (CMS). The LOS for each HFH was computed as the number of days between hospital admission and discharge. A 30-day HF readmission was identified in the MarketScan® databases as any all-cause hospital admission occurring within 30 days of discharge from a HF hospitalization.

### Statistics

Baseline characteristics were compared between noAVB and cAVB patients meeting inclusion criteria. Continuous variables, including follow-up duration and age, were compared using a Student’s *t* test or Mann-Whitney test if the distribution was not normal. Categorical variables, such as sex and baseline comorbidities, were compared using a chi-square (*χ*^2^) test.

The cumulative rate of HFH in the noAVB and cAVB groups was compared using a Poisson regression. Inpatient LOS and 30-day HF readmissions were compared using a Student’s *t* test and chi-square (*χ*^2^) test, respectively. A multivariable Cox proportional hazards model with Andersen-Gill extension and propensity score adjustment was used to evaluate HFH following PM implant. Patients were censored at the time of upgrade to cardiac resynchronization therapy (CRT) or at the end of MarketScan® enrollment. Billing codes used to identify CRT upgrade are outlined in the Supplement (Table S1). The proportional hazards assumption was tested using Schoenfeld residuals and was met. For the outcome of costs associated with HFH, a Mann-Whitney test was used to compare unadjusted and adjusted payments between noAVB and cAVB patients. Statistical significance was determined using α = 0.05.

All analyses were performed on Revolution Analytics Revolution R Enterprise with Open Source R version 3.1.1 or SAS version 9.3. Propensity scores were computed using the LOGISTIC procedure in base SAS.

## Results

### Study cohort

**Fig. 1:**
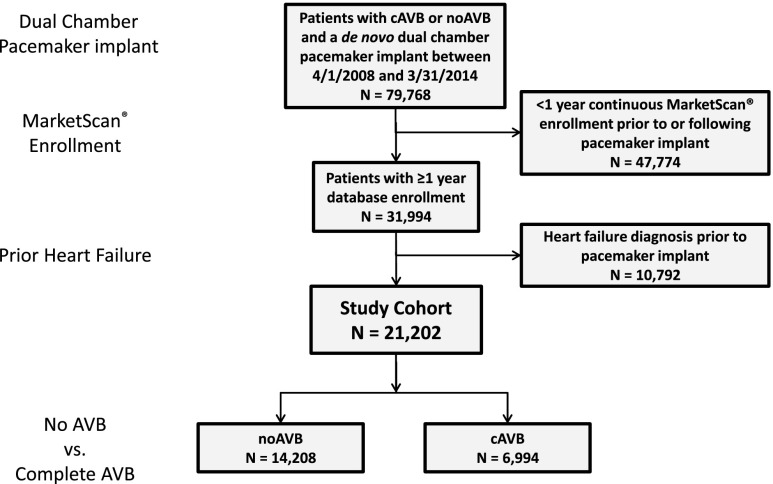
Cohort diagram. Schematic of patients included in the study cohort, including those with no atrioventricular block (noAVB) and those with complete atrioventricular block (cAVB). All patients had a *de novo* dual chamber pacemaker implant between April 1, 2008, and March 31, 2014, and did not have a clinical diagnosis of heart failure in the 1 year prior to implant

The study cohort included 21,202 patients in the MarketScan® databases, of which 14,208 had noAVB and 6994 had cAVB (Fig. 1). The mean age in the study cohort was 74.0 ± 12.6 years and 54% of subjects were male. Baseline characteristics are shown in Table 1. The majority (93%) of patients in the cAVB cohort received a diagnosis of third degree AVB or an AVJ ablation within 1 week of PM implant, most of which (86%) occurred on the same day as PM implant. Overall, 32 noAVB and 61 cAVB patients underwent a CRT upgrade following initial PM implant, accounting for < 1% of the study cohort.

**Table 1:** Baseline characteristics for the study cohort

		noAVB	cAVB	*p* value
		*N* = 14,208	*N* = 6994	
Post-index follow-up duration (years)		2.39 years [1.63, 3.44]	2.27 years [1.58, 3.25]	< 0.001
Sex	Male	7518 (53%)	3996 (57%)	< 0.001
	Female	6690 (47%)	2998 (43%)	
Age (years)		74.0 ± 12.4	73.8 ± 13.1	0.187
Remote monitoring active		5135 (36%)	2564 (37%)	0.470
	2009	2752 (19%)	1267 (18%)	< 0.001
	2010	3893 (27%)	1716 (25%)	
Year of implant	2011	3632 (26%)	1853 (26%)	
	2012	3259 (23%)	1772 (25%)	
	2013	672 (5%)	386 (6%)	
US region	Northeast	2046 (14%)	1391 (20%)	< 0.001
	North Cent.	4600 (32%)	2323 (33%)	
	South	5158 (36%)	2085 (30%)	
	West	2386 (17%)	1185 (17%)	
	Unknown	18 (< 1%)	10 (< 1%)	
Atrial fibrillation		6773 (47.7%)	1433 (20.5%)	< 0.001
VT/VF		697 (4.9%)	254 (3.6%)	< 0.001
Coronary artery disease		6679 (47.0%)	3077 (44.0%)	< 0.001
Hypertension		10,535 (74.1%)	5222 (74.7%)	0.429
Cerebrovascular disease		4111 (28.9%)	1719 (24.6%)	< 0.001
Diabetes		3377 (23.8%)	2058 (29.4%)	< 0.001
Valve disease		4420 (31.1%)	2438 (34.9%)	< 0.001
Peripheral vascular disease		2424 (17.1%)	1177 (16.8%)	0.686
Chronic pulmonary disease		2904 (20.4%)	1458 (20.8%)	0.502
Chronic kidney disease		1245 (8.8%)	642 (9.2%)	0.329
Rheumatic disease		415 (2.9%)	262 (3.7%)	0.002
Peptic ulcer disease		222 (1.6%)	96 (1.4%)	0.313
Liver disease		426 (3.0%)	230 (3.3%)	0.269
Hypothyroidism		2340 (16.5%)	1076 (15.4%)	0.045
Cancer		1772 (12.5%)	998 (14.3%)	< 0.001
Dementia		569 (4.0%)	237 (3.4%)	0.030
Depression		1109 (7.8%)	476 (6.8%)	0.010
AIDS/HIV		8 (0.1%)	5 (0.1%)	0.901
Hemiplegia/paraplegia		149 (1.0%)	59 (0.8%)	0.177
Obesity		607 (4.3%)	362 (5.2%)	0.003

Data reported as count (%), median [interquartile range], and mean ± standard deviation. Continuous variables were compared using a Student’s *t* test or Mann-Whitney test for normal and nonnormal distributions, respectively. Categorical variables were compared using a chi-square (*χ*^2^) test

### HF hospitalizations following PM implant

**Fig. 2:**
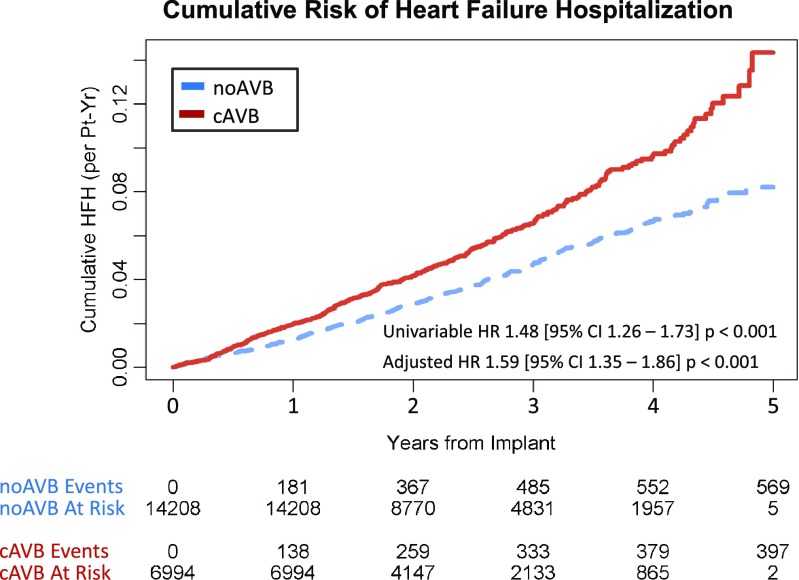
Cumulative risk of heart failure hospitalization following pacemaker implant. Heart failure hospitalizations (HFHs) following pacemaker implant in patients with cAVB versus noAVB. Propensity score adjusted for age, sex, remote monitoring status, US region, year of implant, and 20 baseline comorbidities assessed in the year prior to implant. cAVB complete atrioventricular block, CI confidence interval, HFH heart failure hospitalization, HR hazard ratio, noAVB no atrioventricular block

**Fig. 3:**
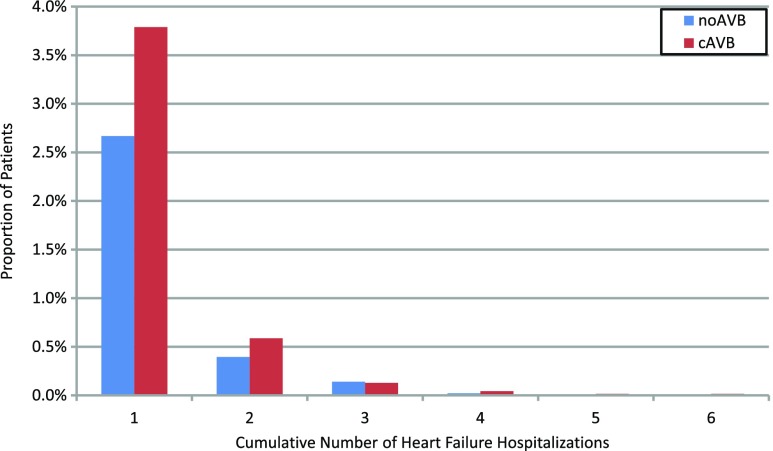
Distribution of number of heart failure hospitalizations following pacemaker implant. Cumulative number of HFH following pacemaker implant in the noAVB and cAVB groups. cAVB complete atrioventricular block, HFH heart failure hospitalization, noAVB no atrioventricular block

Over a median 2.35 [IQR 1.62, 3.39] years of follow-up, 459 noAVB (3.2%) and 320 cAVB (4.6%) patients were hospitalized for HF (*p* < 0.001). The unadjusted rate of HFH was significantly higher for patients with cAVB (2.28 [95% CI 2.06–2.51] per 100 pt-years) compared to those with noAVB (1.55 [95% CI 1.43–1.69] per 100 pt-years) (*p* < 0.001, Table 2). Patients with cAVB were associated with a significantly increased risk of cumulative HFH (adjusted HR 1.59 [95% CI 1.35–1.86], *p* < 0.001, Fig. 2). However, the mean LOS (noAVB 5.1 ± 8.0 days; cAVB 4.6 ± 4.7 days; *p* = 0.181) and the rate of 30-day HF readmissions (noAVB 4.9%; cAVB 5.3%; *p* = 0.914) were not different between groups, indicating that the severity of each HFH was similar for noAVB and cAVB patients (Table 2). Interestingly, the subset of patients with commercial insurance experienced 30-day HF readmission rates of 12.9% overall, with no difference between noAVB and cAVB patients (*p* = 1.000). The 30-day HF readmission rate for those with Medicare Supplemental insurance was 4.2%, similarly with no difference between AVB groups (*p* = 1.000). The majority of hospitalized patients were hospitalized just one time for HF, with a range of 0–6 total HFH over the duration of follow-up (Fig. 3). Only three patients in the entire study cohort (one noAVB and two cAVB) experienced greater than four HFH following PM implant.

**Table 2:** Cumulative HFH rates and unadjusted HFH payments for noAVB and cAVB patients following pacemaker implant

Total study cohort			
	noAVB	cAVB	*p* value
	*N* = 14,208	*N* = 6994	
Number of patients with HFH	459 (3.2%)	320 (4.6%)	< 0.001
HFH event rate (per 100 pt-year)	1.55 95% CI 1.43–1.69	2.28 95% CI 2.06–2.51	< 0.001
30-day HF readmission rate	4.9%	5.3%	0.914
Length of stay (days)	5.1 ± 8.0	4.6 ± 4.7	0.181
Median payment per HFH	$8671 [$5859, $13,441]	$9211 [$5990, $16,796]	0.265
Mean payment (per pt-year)	$241 ± $2624	$397 ± $3554	< 0.001
Commercial insurance			
	noAVB	cAVB	*p* value
	*N* = 3383	*N* = 1843	
Number of patients with HFH	34 (1.0%)	45 (2.4%)	< 0.001
HFH event rate (per 100 pt-years)	0.47 95% CI: 0.33–0.65	1.27 95% CI: 0.96–1.65	< 0.001
30-day HF readmission rate	13.2%	12.7%	1.000
Length of stay (days)	5.8 ± 6.2	6.2 ± 6.8	0.770
Median payment per HFH	$10,620 [$7063, $17,247]	$13,070 [$9271, $22,427]	0.242
Mean payment (per pt-year)	$98 ± $1802	$292 ± $2799	< 0.001
Medicare Supplemental			
	noAVB	cAVB	*p* value
	*N* = 10,825 (76%)	*N* = 5151 (74%)	
Number of patients with HFH	425 (3.9%)	275 (5.3%)	< 0.001
HFH event rate (per 100 pt-years)	1.86 95% CI: 1.70–2.02	2.61 95% CI 2.34–2.90	< 0.001
30-day HF readmission rate	4.3%	4.1%	1.000
Length of stay (days)	5.1 ± 8.1	4.3 ± 4.2	0.070
Median payment per HFH	$8341 [$5772, $13,235]	$8242 [$5672, $14,512]	0.751
Mean payment (per pt-year)	$286 ± $2831	$435 ± $3787	< 0.001

Top panel shows overall study cohort, middle panel only patients with commercial insurance, and bottom panel only patients with Medicare Supplemental insurance plans. Values reported as count (%), median [interquartiles], and mean ± standard deviation

*cAVB* complete atrioventricular block, *CI* confidence interval, *HFH* heart failure hospitalization, *noAVB* no atrioventricular block

### Payments associated with HF hospitalizations following PM implant

**Fig. 4:**
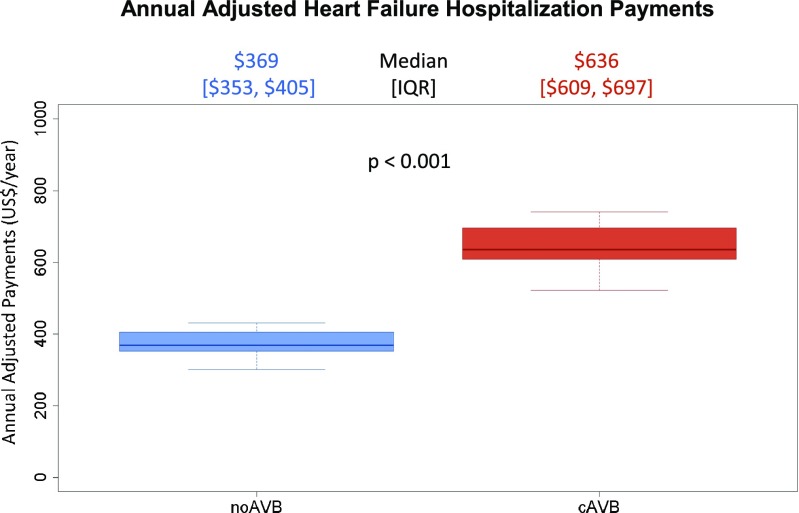
Annual adjusted heart failure hospitalizations payments following pacemaker implant. Results of a two-part model showing the predicted annual payments associated with HFH for patients with noAVB and cAVB. cAVB complete atrioventricular block, HFH heart failure hospitalization, noAVB no atrioventricular block

Patients with noAVB were associated with 42% lower annual adjusted HFH payments compared to those with cAVB (*p* < 0.001, Fig. 4). Similarly, the unadjusted mean payments per pt-year were significantly reduced for patients with noAVB (Table 2). Importantly, the payments per hospitalization were not different between the two groups (Table 2), indicating that the overall cost reduction was driven by the fewer number of patients hospitalized in the noAVB group.

Patients in the study cohort that were enrolled in commercial insurance plans were younger than those covered by Medicare Supplemental insurance plans (56.1 ± 8.7 and 79.8 ± 7.0 years, respectively; *p* < 0.001) and experienced lower rates of baseline atrial fibrillation (28 and 42%; *p* < 0.001), coronary artery disease (36 and 49%; *p* < 0.001), and hypertension (63 and 78%; *p* < 0.001). Remote monitoring utilization was also higher for commercially insured patients (42 versus 34%; *p* < 0.001). While HFH was generally less common for patients with commercial versus Medicare Supplemental insurance, patients with cAVB were associated with higher rates of HFH and associated payments compared to those with noAVB, regardless of the type of insurance (Table 2). The median payment per HFH was not different between patients with cAVB versus noAVB in both the commercial (*p* = 0.242) and Medicare Supplemental (*p* = 0.751) groups, although those hospitalizations covered by commercial insurance were generally higher in cost compared to hospitalizations covered by Medicare (Table 2).

## Discussion

Using data from a large nationwide claims database, we find that patients without an antecedent history of HF and with a presumed high burden of RV pacing are associated with a significantly heightened risk of HF hospitalization and related healthcare costs. We have previously evaluated clinical outcomes in this same population and have shown that complete AVB is associated with increased risk of new onset HF, which appears to develop quite soon after pacemaker implantation [1]. In the current study, we illustrate that over a median of nearly 2½ years of follow-up, 4.6% of patients with complete heart block were hospitalized with HF, which was 44% greater than the 3.2% rate observed in patients without complete AV block. Taken together, these analyses suggest that the increase in new onset HF associated with RV pacing in turn leads to a 59% increase in cumulative hospitalizations and a 72% increase in heart failure hospitalization-related payments.

Hospitalization constitutes the major contributor to the expense related to the care of HF patients [6]. Higher costs can occur if patients experience more severe decompensations of HF and/or a greater number of hospitalizations during follow-up. In our study, we show that although patients with cAVB undergoing pacemaker implantation had more hospitalizations, the length and cost per hospitalization did not vary between patients with and without cAVB. This suggests that costs are being driven by the number of hospitalizations and not the severity of HF. In fact, it was the first hospitalization for HF following pacemaker implantation that occurred more commonly in cAVB patients.

The short- and long-term adverse impact of right ventricular pacing is now well understood. There are changes in electrical and mechanical activation, alterations in metabolism and perfusion, adverse atrial and ventricular remodeling, changes in hemodynamics, and changes in mechanical function [2]. Although in individual patients each of these changes has been observed either alone or in combination, it has been more difficult to ascertain the adverse impact of pacing in cohorts of patients followed over time.

### Prior studies (Table 3)

Zhang et al. reported 304 patients who underwent ventricular pacing for second or third-degree AV block [13]. Patients were excluded if their ejection fraction was < 50% prior to pacemaker implantation, if they had an existing diagnosis of HF, and if ventricular pacing occurred < 90% of the time during follow-up. After a median follow-up of 7.8 years, 26% of patients developed new-onset HF. Of note, 18% of the cohort underwent single-chamber ventricular pacing and these patients were much more likely to develop HF. Our study was limited to inpatient HFH for patients who received a dual-chamber pacemaker, which likely explains our lower observed incidence of HFH. Importantly, a previous publication from our group on the same cohort analyzed in the current study found that 28% of patients with cAVB received a clinical diagnosis of HF in the inpatient or outpatient setting during the 4 years following pacemaker implant, which aligns well with the study by Zhang et al. [1]. Ebert et al. enrolled 991 patients who underwent pacemaker implantation for either AV block (*n* = 500) or sinus node disease (*n* = 491); the cohort included patients with normal (> 55%, *n* = 791) or mildly reduced (41–55%, *n* = 200) ejection fraction [3]. Over a follow-up period of 44 months, 17% of the cohort died and 6% experienced a ≥ 2 LVEF category deterioration. The indication for pacing and baseline ejection fraction had no impact on outcome. Again, 14% of the cohort underwent only a single-chamber device and heart failure was not a measured outcome variable, unless it necessitated upgrade to a CRT device.

**Table 3:** Prior studies that have sought to assess the adverse impact of right ventricular pacing

Patient cohort	Number of patients	Follow-up	Primary endpoint	Result
Patients with AV block, no prior history of HF, who were RV paced > 90% of the time [13]	304	94 months	Prevalence and clinical predictors for development of HF	26% of patients developed HF, which was associated with increased cardiovascular mortality
Patients with complete AV block and a dual chamber pacemaker, no prior history of HF [1]	21,202	48 months	Clinical diagnosis of HF during an inpatient or outpatient encounter, as reflected by billing codes	28% of patients developed HF.
				The incidence was higher in the first 6 months post-implant.
				Younger individuals and those with a history of AF experienced the highest risk of new HF
Patients with baseline normal (> 55%, *n* = 791) or mildly reduced (41–55%, *n* = 200) LVEF	991	44 months	All-cause mortality and deterioration of LV function ≥ 2 LVEF categories at last follow-up	Death from any cause occurred in 17% and deterioration of LV function ≥ 2 LVEF categories in 6% patients.
Follow-up who underwent PPM implantation for AV block (*n* = 500) or sinus node disease (*n* = 491). [3]				There was no significant difference in outcome between patients with AV block and sinus node disease.
Patients with normal LVEF who were RV paced > 20% of the time [4]	257	40 months	Development of a PICM (≥ 10% decrease in LVEF resulting in LVEF < 50%)	~ 20% likelihood of developing a PICM
Consecutive patients with complete heart block and LVEF > 50% under-going PPM implantation [5]	823	52 months	Development of a PICM CRT upgrade or LVEF ≤ 40%)	12% likelihood of developing a PICM

*AF* atrial fibrillation, *AV* atrioventricular, *CRT* cardiac resynchronization therapy, *HF* heart failure, *LVEF* left ventricular ejection fraction, *PICM* pacing-induced cardiomyopathy, *PPM* permanent pacemaker, *RV* right ventricular

Two additional studies have examined the development of a pacing induced cardiomyopathy (PICM) following right ventricular pacing. The first study evaluated 1750 consecutive patients who underwent pacemaker implantation; a study cohort of 257 patients was identified who underwent single or dual chamber pacemaker implantation, had normal LV function at baseline, had ≥ 20% RV pacing, and had a repeat echocardiogram ≥ 1 year following pacemaker implantation [4]. PICM was defined as ≥ 10% decline in LVEF, resulting in a LVEF < 50%. During a mean follow-up of 3.3 years, ~ 20% of patients developed a PICM. Whether this resulted in HF or other adverse clinical events was not assessed. The second study evaluated consecutive patients with complete heart block and LVEF > 50% who underwent pacemaker implantation [5]. PICM was defined as CRT upgrade or a decline in LVEF to ≤ 40%. During a mean of 4.3 years, 12% of the cohort developed a PICM. Patients with ≥ 20% RV pacing were at significantly greater risk for developing a PICM. Our study is unique given the large sample size, inclusion of only patients with a dual chamber pacemaker, and use of claims data to identify all patients with hospitalizations related to new-onset HF and healthcare costs associated with the care of these patients.

Heart failure is the most common reason for hospitalization among the elderly; although patients with HF represent only 14% of the overall Medicare population, they account for 43% of Medicare spending [14]. It has been estimated that a diagnosis of HF is associated with annual costs of $8500 per patient; three quarters of the total costs are associated with hospital admissions, in-hospital treatment, and patient care in nursing homes [15]. Importantly, costs are high at the time of initial diagnosis, likely reflecting that the initial diagnosis is often made while patients are hospitalized [6]. Thus, it is important to identify patients who may be at risk for developing HF. Our study shows that one at risk population is comprised of patients who undergo dual-chamber pacemaker implantation for management of complete heart block. These patients were at significantly greater risk of being hospitalized, which resulted in increased healthcare payments to manage these patients. This suggests that strategies to prevent development of heart failure in pacemaker patients may have significant positive implications to the healthcare system. To date, there is interest in determining whether selective conduction system pacing or biventricular pacing may mitigate the adverse clinical effects of right ventricular pacing in patients with advanced heart block.

### Limitations

The major limitations of this study are that, given the nature of claims data, we do not have information about ejection fraction (either at baseline or over follow-up) and thus cannot distinguish between heart failure with preserved or reduced ejection fraction. Additionally, we presume the dual chamber pacing in patients with complete heart block will result in high burden of ventricular pacing; by nature of this analysis, we lack information about actual percentage of pacing delivered in these patients. As a result, we are also unable to determine whether there is a threshold degree of pacing that results in development of heart failure. Finally, mortality is not available in the MarketScan® dataset used for this analysis, so could not be evaluated.

## Conclusions

In a large, national cohort of patients undergoing pacemaker implantation, those with a diagnosis of complete heart block (who likely have a high burden of RV pacing) experienced a significantly increased risk of HF hospitalization. This was associated with higher healthcare payments for the care of these patients. Future efforts need to identify patients at greatest risk and develop strategies to mitigate the need for hospitalization in these patients, as this remains a potent driver to overall healthcare costs.

## Electronic supplementary material

Table S1 (DOCX 16 kb).
