# Supplementary material for: Increased healthcare utilization associated with complete atrioventricular block in pacemaker patients
Source: J Interv Card Electrophysiol. 2018 Feb 28;51(3):221–8. doi: 10.1007/s10840-018-0336-0 (PMC5902523; doi:10.1007/s10840-018-0336-0)
Supplement: Supplementary file 1 — (DOCX 16 kb). [file 10840_2018_336_MOESM1_ESM.docx]

Table S1. Insurance Claims Codes Used for Patient Characterization

| Condition | Codes |
| --- | --- |
| Third Degree AVB | 426.0 |
| Other AVB | 426.10-426.13 |
| Heart Failure | 398.91,402.01,402.11,402.91,404.01,404.03,404.11,404.13,404.91,404.93, 425.4-425.9,428.x |
| Atrial Fibrillation | 427.31 |
| VT/VF | 427.1,427.4x |
| Coronary Artery Disease | 410.x-414.x,429.2,V45.81 |
| Hypertension | 401.x-405.x,437.2 |
| Stroke | 433.x1,434.x1,435.x,436,438.x |
| Diabetes | 250.X |
| Valvular Heart Disease | 394.x-397.x,424.x,746.x,V42.2,V43.3, |
| Cerebrovascular Disease | 362.34,430.x-438.x |
| Peripheral Heart Disease | 093.0,437.3,440.x,441.x,443.1-443.9,447.1,557.1,557.9,V43.4 |
| Chronic Pulmonary Disease | 416.8,416.9,490.x-505.x,506.4,508.1,508.8 |
| Chronic Kidney Disease | 403.1,403.11,403.91,404.02,404.03,404.12,404.13,404.92,404.93,582.x,583.0-583.7,585.x,586,588.0,V42.0, V56.x |
| Rheumatic Disease | 446.5,710.0-710.4,714.0-714.2,714.8,725.x |
| Peptic Ulcer Disease | 531.x-534.x |
| Liver Disease | 070.22,070.23,070.32,070.33,070.44,070.54,070.6,070.9,456.0-456.1,570.x,571.x,572.2-572.8,573.3,573.4,573.8, 573.9,V42.7 |
| Hypothyroidism | 240.9,243.x,244.x,246.1,246.8 |
| Cancer | 140.x-172.x,174.x-194.x,195.0-195.8,196.x,197.0-197.8,198.0-198.8x,1990-1992,200.x-208.x,238.6 |
| Dementia | 290.x,331.0,331.2 |
| Depression | 300.4,309.x,311 |
| Hemiplegia/Paraplegia | 334.1, 342.00-342.92,343.x,344.1,344.2,344.5,344.9 |
| AIDS/HIV | 042 |
| Obesity | 278.00 |

Claims codes used for diagnoses and procedures collected across all available fields (up to fifteen) in the MarketScan® inpatient and outpatient encounters.
